# Exercise Related Respiratory Problems in the Young—Is It Exercise-Induced Bronchoconstriction or Laryngeal Obstruction?

**DOI:** 10.3389/fped.2021.800073

**Published:** 2022-01-03

**Authors:** Maria Vollsæter, Trine Stensrud, Robert Maat, Thomas Halvorsen, Ola Drange Røksund, Astrid Sandnes, Hege Clemm

**Affiliations:** ^1^Department of Paediatrics and Adolescent Medicine, Haukeland University Hospital, Bergen, Norway; ^2^Department of Clinical Science, Section for Paediatrics, University of Bergen, Bergen, Norway; ^3^Department of Sports Medicine, Norwegian School of Sport Sciences, Oslo, Norway; ^4^Department of Otorhinolaryngology, Saxenburgh Medical Center, Hardenberg, Netherlands; ^5^Faculty of Health and Social Sciences, Bergen University College, Bergen, Norway; ^6^Department of Internal Medicine, Innlandet Hospital Trust, Gjøvik, Norway

**Keywords:** exercise, shortness breath, young, exercise-induced bronchoconstriction (EIB), exercise-induced laryngeal obstruction (EILO)

## Abstract

Complaints of breathlessness during heavy exercise is common in children and adolescents, and represent expressions of a subjective feeling that may be difficult to verify and to link with specific diagnoses through objective tests. Exercise-induced asthma and exercise-induced laryngeal obstruction are two common medical causes of breathing difficulities in children and adolescents that can be challenging to distinguish between, based only on the complaints presented by patients. However, by applying a systematic clinical approach that includes rational use of tests, both conditions can usually be diagnosed reliably. In this invited mini-review, we suggest an approach we find feasible in our everyday clinical work.

## Introduction

Exercise related breathing complaints are common in children and adolescents, and a scenario all clinicians must be prepared to encounter. The experience of breathlessness or dyspnea during heavy exercise is a subjective feeling, and may often be difficult to verify through objective tests. Dyspnea during exercise may be caused by numerous respiratory or non-respiratory factors. This was nicely exemplified by a study by Abu Hasan et al. (1) where exercise induced breathing complaints were present in 142 included young adults, and observed in 82% during exercise testing. Importantly, normal physiological exercise limitations were demonstrated in 52%, exercise induced bronchoconstriction (EIB) in 8%, and symptoms of exercise induced laryngeal obstruction (EILO) in 9%. As most patients in fact had neither EIB nor EILO, the study supports a broader view on other causes of exercise induced breathing complaints, like dysfunctional breathing patterns (2), low aerobic capacity or other. This and other studies (3) have underlined the heterogeneous nature of exercise induced complaints, and provided support to guidelines recommending that exercise induced breathing complaints should be evaluated with objective tests (4) and that EILO might be just as frequent as EIB (5, 6).

Asthma is common in children and adolescents, and a well-established cause of EIB, with current prevalence rates 5–20% in the general population (7). Up to 90% of patients with symptomatic asthma are reported to have some degree of EIB (8). Untreated EIB may lead to exercise intolerance and reduced participation in play and sport (9). Despite guidelines prescribing objective test methods (4), studies suggest that asthma and EIB are often diagnosed simply based on wheeze as presenting symptoms, which is a highly questionable strategy (3, 10–12).

The purpose of this mini-review is to outline a practical diagnostic approach to children and adolescents presenting with exercise induced breathing complaints, focusing on separating EIB from EILO.

### Exercise and Breathing in General

Exercise increases the metabolic demands of the body and leads to increased pulmonary ventilation. The increased ventilation is accomplished mainly by greater tidal volumes at low intensity exercise, while at higher intensities, the breathing frequency increases alongside a decrease of particularly the expiratory time (13, 14). The minute ventilation may rise 30 times over resting values at peak exercise (13, 15). When the minute ventilation rises during exercise, most people shift from nasal to oral breathing, to reduce airflow recistance (16). Active opening of the mouth tends to widen the laryngeal opening, allowing for higher airflow with less increase of laryngeal resistance (17).

High intensity exercise imposes stress on the respiratory system. Ideally, breathing is regulated to maximize the ability to perform. Anatomy, physiological capacities, and the state of the cardiopulmonary system are all important factors for performance. Various exercise modalities/sports may challenge ventilation, and optimal conditions for diaphragmatic and thoracic expansion depend on body posture and breathing frequency (18, 19). The larynx plays a role in some exercise modalities, where closure of the glottis facilitates elevation of thoracic and abdominal pressures (20).

## Exercise Induced Bronchoconstriction

Atopic asthma often starts in childhood and is characterized by allergies and bronchial mucosal eosinophilic inflammation. The patient may experience chest tightness, dyspnea, wheeze and cough during exercise, and also when exposed to allergens or airway infections (21). Asthma is a clinical diagnosis based on a combination of symptoms and objective findings. Global Initiative of Asthma (GINA) defines asthma as symptoms that vary in time and intensity, alongside variable expiratory airway obstruction (22). EIB is present in a great proportion of young people with untreated or under-treated asthma (8), but is also a feature observed in athletes with or without previous asthma (4, 23). In athletes, EIB is often a result of years of high-intensity exercise in unfavouralbe environmental conditions, such as cold air, polluted air or chlorine (24–26). EIB is suggested characterized by neutrophilic inflammation, or a combined neutrophilic and eosinophilic inflammation (27). In sports, EIB is seemingly related to prolonged mechanical, osmotic and thermic stress, with subsequent destruction of airway epithelia (28). These findings are related to the time spent exercising in harsh conditions, and also to the timing of the testing, whether this is in close relation to a period of intensive training or competition (25). Increased bronchial parasympathetic tone has been reported in skiers, suggesting that the parasympathetic nervous system is implicated in the development of EIB in athletes (29, 30).

EIB leads to respiratory symptoms during or after exercise, like chest tightness, dyspnea, cough or secretions. However, exercise related breathing complaints are poorly associated with objective findings, and tests are therefore required to diagnose both asthma and EIB (3, 31). Spirometry with reversibility measurements after inhaled beta2 agonists or ipratropium bromide is a minimum (32). Measurement of exhaled nitric oxide might reveal eosinophilic inflammation, and there are different bronchial provocation tests to diagnose bronchial hyperreactivity to direct or indirect stimuli. A direct stimulus is metacholine, a parasympatic agent acting directly on muscarine receptors on smooth mucle cells in the airway wall, leading to bronchoconstriction (33). Indirect stimuli are based on the theory that fluid and heatloss from the airways during high minute ventilation leads to bronchoconstriction (34). Exercise-tests and eucapnic voluntary hyperventilation (EVH) tests are the classical examples of such indirect stimuli, but inhaled mannitol or hypertonic saline are also based on these same principles (35). EVH is now valued as the most sensitive test available (36, 37), however EIB-test with inhaled cold or dry air, is more specific (35, 38).

EIB is a response to increased ventilation induced by high intensity exercise, with expiratory symptoms, usually peaking 3–15 mins after ending exercise (39). This response contrasts that caused by upper airway obstruction, that typically peak during exercise or just after stopping and presents on inspiration. The literature indicates that EIB is overdiagnosed in patients with EILO (40, 41).

## Exercise Induced Laryngeal Obstruction

Exercise induced laryngeal obstruction (EILO) refers to inappropriate transient adduction of the structures in the larynx during exercise and is a common cause of exercise induced respiratory symptoms. The nomenclature proposed by the joint “Task Force on Inducible Laryngeal Obstructions” (42) underlines that EILO can occur on two laryngeal levels, the supraglottic and the glottic (vocal fold) level.

EILO is a clinical diagnosis, where the exercise induced medialisation of laryngeal structures leads to inspiratory airflow obstruction during exercise, usually characterized by typical breathing complaints. The critical point of obstruction of the larynx required to produce symptoms in any given individual is difficult to assess, and probably depends on the timing of the breathing cycle, the airflow, the absolute size of the larynx or other individual differences (43). Airflow through the larynx increases with increased exercise intensity, and as increased flow through a tube inevitably sets up a negative pressure within that tube, this mechanism may be involved in the laryngeal adduction or collapse observed in EILO patients. Depending on the airflow velocity, turbulence, laryngeal architecture, and the strength of the supporting structures, the larynx of a patient with EILO will eventually yield to the negative pressure (17, 44–46). The normal or optimal relations between body size, ventilatory requirements, and the absolute size of the laryngeal aperture remain to be shown (47).

EILO is usually characterized by respiratory difficulties during inspiration, sometimes accompanied by stridor-like breath sounds. Some EILO patients develop hyperventilation or panic reactions with increasing ventilatory requirement parallel to increasing exercise intensity (43, 48). Many individuals have difficulties describing their symptoms, and inspiratory symptoms might associate to a wide spectrum of structural and functional abnormalities; therefore, testing is necessary (5, 6, 40, 49, 50). The gold standard for diagnosing EILO is the continuous laryngoscopy during exercise (CLE-test) (48). The test will reveal the level and starting point of obstruction, as well as the degree, scored by the CLE score (51), information which may guide further treatment approach (52, 53). Spirometry should be included in the diagnostic work-up in all patients with exercise related breathing complaints, however, spirometry patterns are not diagnostic for EILO, neither are bronchial hyperreactivity tests (54).

Like with EIB, environmental factors may contribute in EILO. There are reports of EILO worsening in cold and humid conditions (55, 56), as well as being more frequent among athletes participating in outdoor activities (57).

### EIB or EILO?

EIB and EILO both present as exercise related breathing complaints in the young. EIB is a diagnosis easily resorted to, possibly because of the high degree of awareness among physicians and patients. EILO is a less well-known diagnosis, and therefore possibly less likely to be considered. Ersson et al. found an estimated prevalence of EIB in 23% and EILO in 8% of adolescents attending first year of sports high school in Sweden, underlining the importance of EILO as differential diagnosis to EIB (58). In two other Scandinavian studies of unselected children with exercise related complaints, the EILO prevalence was 5% and 7.5% (5, 6). As for patient demographics, EILO is seemingly most common in young females (59, 60). EILO symptoms are often confused with symptoms of EIB (21, 61, 62), and retrospective reports indicate that asthma medication has often been prescribed to patients later diagnosed with EILO (63, 64). In a study of 151 EILO patients, 85% had used asthma treatments, 64% of these with no effect on exercise related symptoms (40). EIB and EILO may also coexist, leading to confusion regarding the effect of asthma treatment (6, 57, 63–66). Treating EILO as asthma might lead to overmedication, frustration, and eventually cessation of sports activities in youngsters (40).

Patients with exercise relatd breathing difficulties should be tested by means of an exercise test, and ideally with the particular type of exercise and under the particular environmental conditions that led to their symptoms (31). In routine clinical work, standardized exercise tests are usually applied, due to practical challenges setting up customized tests and contidions, and problems related to test-retest reproducibility (67, 68). Close observation and thorough reporting of symptom development during any exercise test is essential.

**Figure 1:**
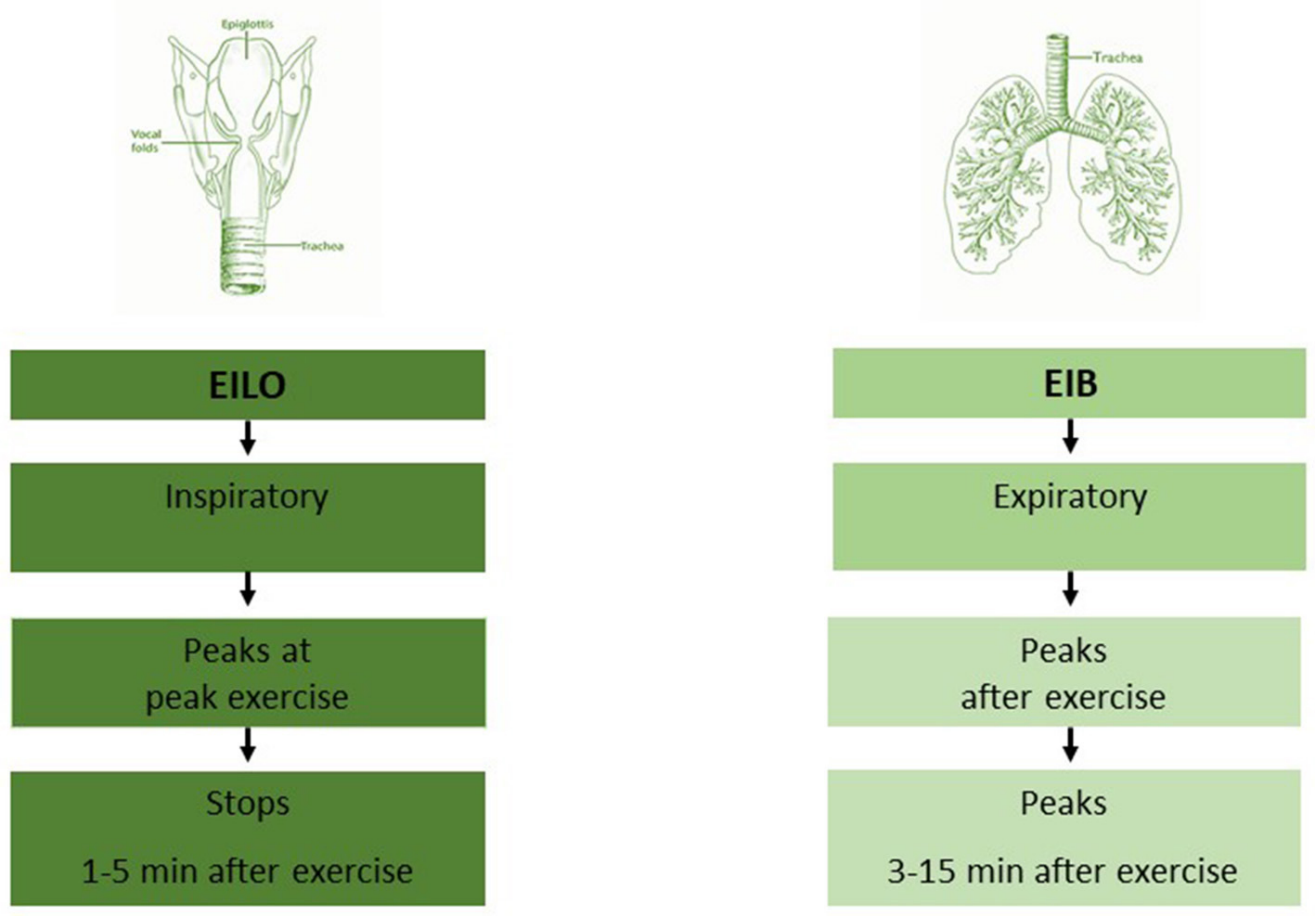
Work-up should be customized to the level of complaints, but always involve a thorough symptom description. Spirometry, and a standardized exercise test for *exercise induced bronchoconstriction (EIB) are logical second steps. The EIB test should always include the test-leaders thorough description of breathing patterns during and after the test, and a patient verification that the symptoms perceived during the test were similar to those that in-cited the work-up. Patients with classical symptoms of EILO observed during an exercise test may not need a CLE test, depending on the clinical situation. Persistent symptoms without findings must incite further work-up. **Diagnostic tests for BHR might include Spirometry with reversibility, PD20, EVH, Mannitol or hypertonic saline, depending on the clinical situation and department policy. EIB, exercise induced bronchoconstriction; EILO, exercise induced laryngeal obstruction; BHR, bronchial hyperresponsiveness; CLE, continuous laryngoscopy exercise test; CPET, cardiopulmonary exercise test. Modified from Paediatric Respiratory reviews (47).

Most studies of both EILO and EIB include adolescents (5, 6, 69). During these years the intensity of physical exercise often increases, and they approach their ventilation limits more often than earlier. As the age-span for symptoms of both conditions is wide, it is important that we provide a test-battery suited for all ages. Both EIB and EILO can occur at a young age, well documented in EIB (70), but more scarcely in EILO (71). Alternative and appropriate means of testing should be considered in the youngest patients, as well as in patients of all ages with various impairments, as both spirometry and treadmill running or cycling might be challenging or impossible. Other forms of strenuous activity can be applied to elicit the symptoms (70, 72), an appropriate heart rate should be achieved. This may be accompanied by spirometry, or by close observation of breathing patterns during and after exercise, in order to differentiate between the two conditions EIB and EILO (Figure 1). This observation period needs to be prolonged in order to allow for EIB symptoms to arise, corresponding to the time allowed for repeated spirometries after a standard EIB test. As for EILO, there are currently no appropriate alternatives to the CLE-test.

## A Systematic Diagnostic Approach to Exercise Induced Breathing Complaints

**Figure 2:**
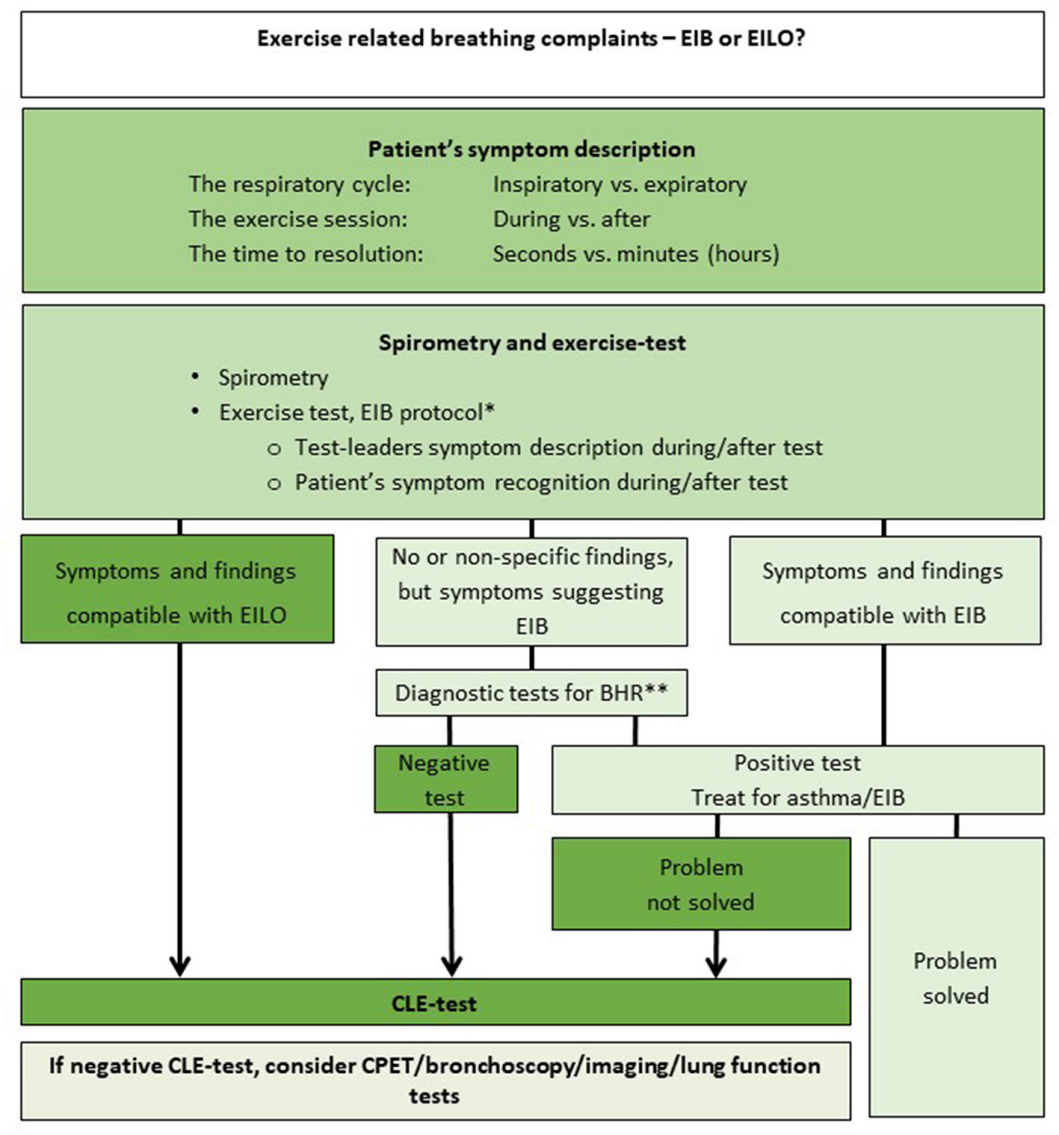
Symptom presentation in the patient with exercise induced breathing complaints, differences between EIB and EILO. EIB, exercise induced bronchoconstriction; EILO, exercise induced laryngeal obstruction. Modified from Paediatric Respiratory reviews (47).

We consider the standardized exercise induced bronchoconstriction (EIB) test to be the first choice and the cornerstone in the diagnostic evaluation of exercise related breathing complaints. The change after exercise in forced expiratory volume in first second (FEV_1_) is the main objective outcome measure of an EIB test; however, of similar importance is the patient's symptoms presentation and breathing patterns during the test. These items should be observed and thoroughly described by the test-leader, and be documented and made available for the physician interpreting the test (Figure 2). The symptoms have to be described during and after exercise, whether they are most prominent at inspiration or expiration, whether they peak at or after maximum ventilation, and the time to symptoms resolve. Also, accompanying symptoms should be described, respiratory sounds, patient reactions and breathing patterns. Video recording of the patient and the symptom presentation during the test, may be considered. The patient could even bring her/his own video of the symptoms, recorded during (or immediately after) exercise, the video should include sound. Whether the observed symptoms are representative for the breathing complaints that led to the referral for testing is of great interest. Of note, dysfunctional breathing (2) or insufficient effort is of great importance. Insufficient intensity might lead to a false negative test, as a high ventilation is required to elicit EIB. The requirement for the patient being able to reproduce symptoms during the test, is absolute for both EIB and EILO tests.

A decline in FEV_1_ of 10% or more after exercise is considered a positive test for EIB, (73) and treatment should be initiated in accordance with recommendations (22). A negative test combined with strong clinical suspicion of EIB should incite more specific tests for bronchial hyperreactivity, a topic which is outside the scope of this article (4).

Inspiratory breathing difficulties observed during an EIB test should lead to suspicion of EILO, and a CLE-test should be considered (42). If EILO is diagnosed, breathing advice and/or other forms of treatment should follow accordingly (47, 55). There are currently no guidelines to recommend an evidence based treatment approach for EILO, and this is evidently an under-reserched area of respiratory medicine.

If the CLE test reveals normal findings and the symptoms suggest inspiratory obstruction, other diagnostic tests should be considered, guided by the nature of the presenting symptoms and their severity. A cardiopulmonary exercise test evaluates cardiopulmonary responses and limitations in relation to exercise, such as low aerobic capacity, heart disease, lung diseases with ventilatory limitations or dysfunctional breathing (2, 74), and measures breathing frequency and tidal volumes, which could be used to objectively assess breathing patterns during exercise in cases where an abnormal breathing patterns have been described.

Bronchoscopy or radiologic imaging of central airways and the thoracic cage could reveal for example tracheobronchomalacia, excessive dynamic airway collapse (75), or inborn vascular malformations that compromise central airways and thereby produce symptoms that are aggrevated by high intensity exercise. We have previously found structural abnormalities in the upper airways in ~10% of patients refered to our institution under the suspicion of EILO (76).

## Conclusion

Exercise related breathing complaints are common in the young. The symptom presentation should be accurately described but cautiously interpreted, as widely different conditions might present with symptoms that are similarly described by patients. Important factors to consider are the timing of the breathing complaints in relation to the exercise, and if the problem is inspiratory or expiratory; i.e., inspiratory stridor or expiratory wheeze, and the time to symptom resolution after exercise. Patients with exercise related breathing difficulties should be tested by means of exercise, ideally with the type of exercise and under the environmental conditions that lead to their symptoms. Spiromtery performed before vs. after exercise and a visual representation of the larynx throughout an ongoing maximal exercise test are the two recommended means to diagnose EIB and EILO, respectively. A thorough symptoms description during the exercise tests is also important, in order to reveal (co)morbidities that may cause or blur the picture, like dysfunctional breathing.

## Author Contributions

MV, TS, RM, TH, OR, AS, and HC made a significant contribution to the conception and the design of the article and of the collection, analysis and interpretation of the data, drafting of the article, and revising it critically for content and final approval of the version to be published. All authors participate in the Upper Airway Group of Haukeland University Hospital and are collectively responsible for the final version of this paper.

## Funding

Major funding institutions: Haukeland University Hospital, University of Bergen and Bergen University College.

## Conflict of Interest

The authors declare that the research was conducted in the absence of any commercial or financial relationships that could be construed as a potential conflict of interest.

## Publisher's Note

All claims expressed in this article are solely those of the authors and do not necessarily represent those of their affiliated organizations, or those of the publisher, the editors and the reviewers. Any product that may be evaluated in this article, or claim that may be made by its manufacturer, is not guaranteed or endorsed by the publisher.

## Abbreviations

CLE-test: continuous laryngoscopy exercise-test
CPET: cardiopulmonary exercise test
EIA: exercise induced asthma
EIB: exercise-induced bronchoconstriction
EIIS: exercise induced inspiratory symptoms
EILO: exercise induced laryngeal obstruction
FEV_1_: forced expiratory flow in the first second
PCA-muscle: the posterior cricoarytenoid muscle
VCD: vocal cord dysfunction.
